# Supplementary material for: SHIELD: a platform for high-throughput screening of barrier-type DNA elements in human cells
Source: Nat Commun. 2023 Sep 12;14:5616. doi: 10.1038/s41467-023-41468-3 (PMC10497619; doi:10.1038/s41467-023-41468-3)
Supplement: Supplementary file 1 — Supplementary Information [file 41467_2023_41468_MOESM1_ESM.pdf]

Supplementary Information for

SHIELD: A platform for high-throughput screening of barrier DNA elements in human cells

Meng Zhang, Mary Elisabeth Ehmann, Srija Matukumalli Aashutosh Girish Boob, David Gilbert, Huimin Zhao*

*Corresponding author. Email: zhao5@illinois.edu (H.Z.)

This file includes:

Supplementary Figures 1-14
Supplementary Tables 1-12
References

Supplementary Excel File S1 (Separate File)

Supplementary Figures

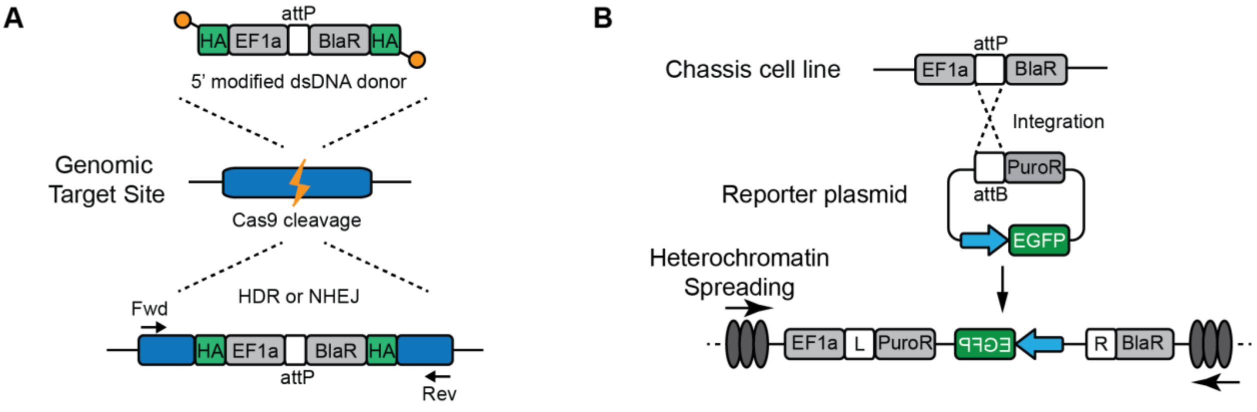

Supplementary Figure 1. Overview of the CRISPR/Cas9-based KI of landing pads and PhiC31 integrase-based reporter plasmid integration. (**A**) The landing pad was inserted at the selected genomic target site following the repair of Cas9-induced double-strand break using the dsDNA as donor. The donor DNA was modified at the 5' ends (see Methods) to improve knock-in efficiency. For the dsDNA donor for target site H1, we included a 48-mer tetO repeat array (~1.5 kb) upstream of the landing pad for visualization purpose. Fwd/Rev: Forward or reverse primers used to screen clones with on-target KI. HA: homology arm. HDR: homology-directed repair. NHEJ: non-homologous end joining. (**B**) The reporter plasmid was integrated at selected loci via the integrase-mediated recombination. *PuroR* was used as the marker to select for on-target integration, whereas *EGFP* was adopted as the reporter gene to track transgene expression at selected loci over time. Blue arrow denotes the promoter that drives *EGFP* expression. L = attL; R = attR. For simplicity, the bGH polyadenylation signal sequence downstream of each gene (*BlaR*, *PuroR*, *EGFP*) is not shown.

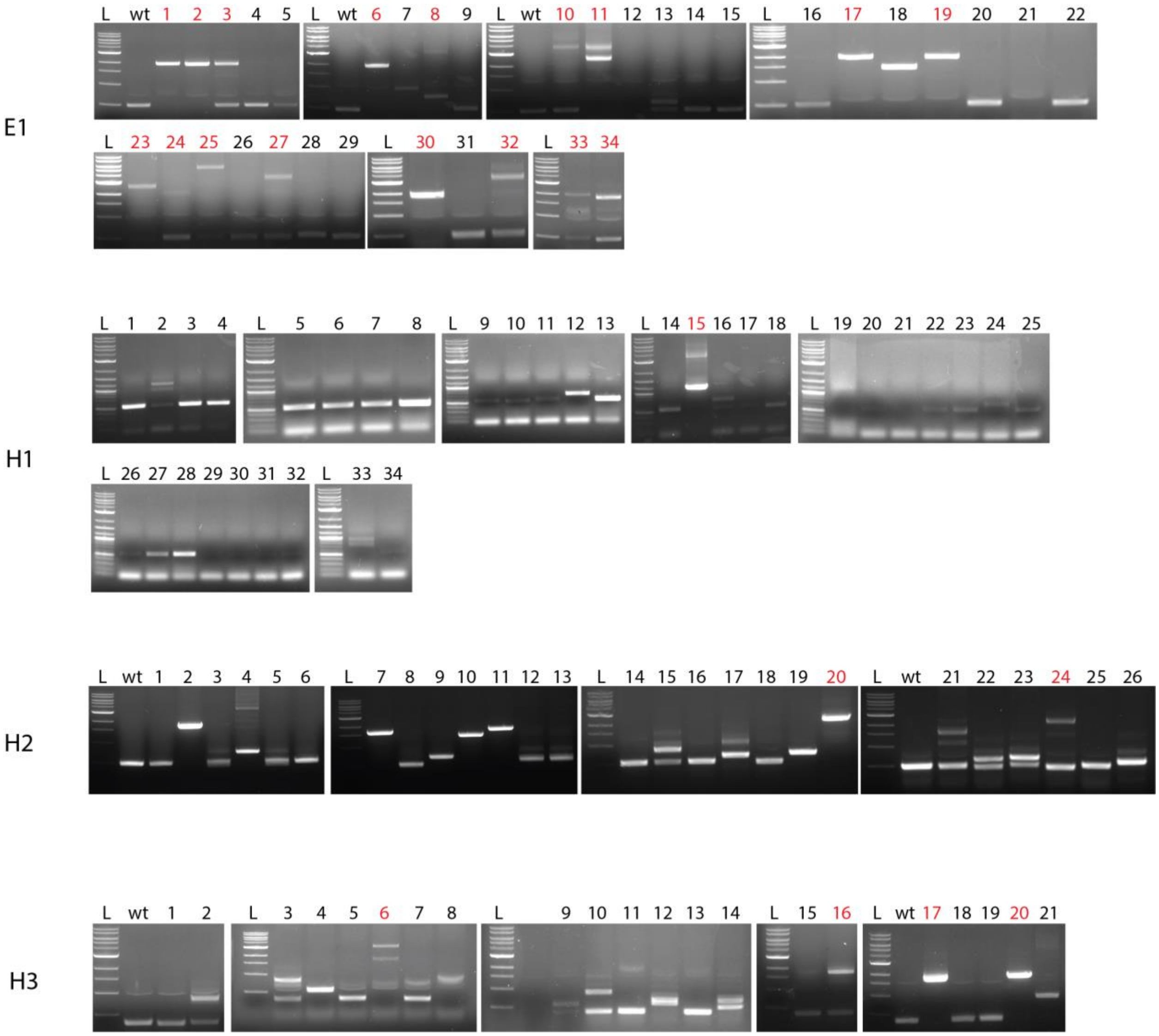

Supplementary Figure 2. Genotyping PCR of individual clones from CRISPR/Cas9-based landing pad insertion at four loci. PCR was performed with Fwd and Rev primers shown in Supplementary Figure 1A (Supplementary Table 1) with genomic DNA isolated from each clone. Each lane is numerically labeled with the corresponding clone number. For simplicity, only clones with PCR products no shorter than the expected length (assuming homologous recombination) were considered positive and are highlighted in red. Expected KI band: E1 (~1.9 kb), H1 (~3.5 kb), H2 (~2 kb), H3 (~1.8 kb). L = ladder (NEB 1 kb DNA ladder), wt = wild-type genomic DNA used as a negative control.

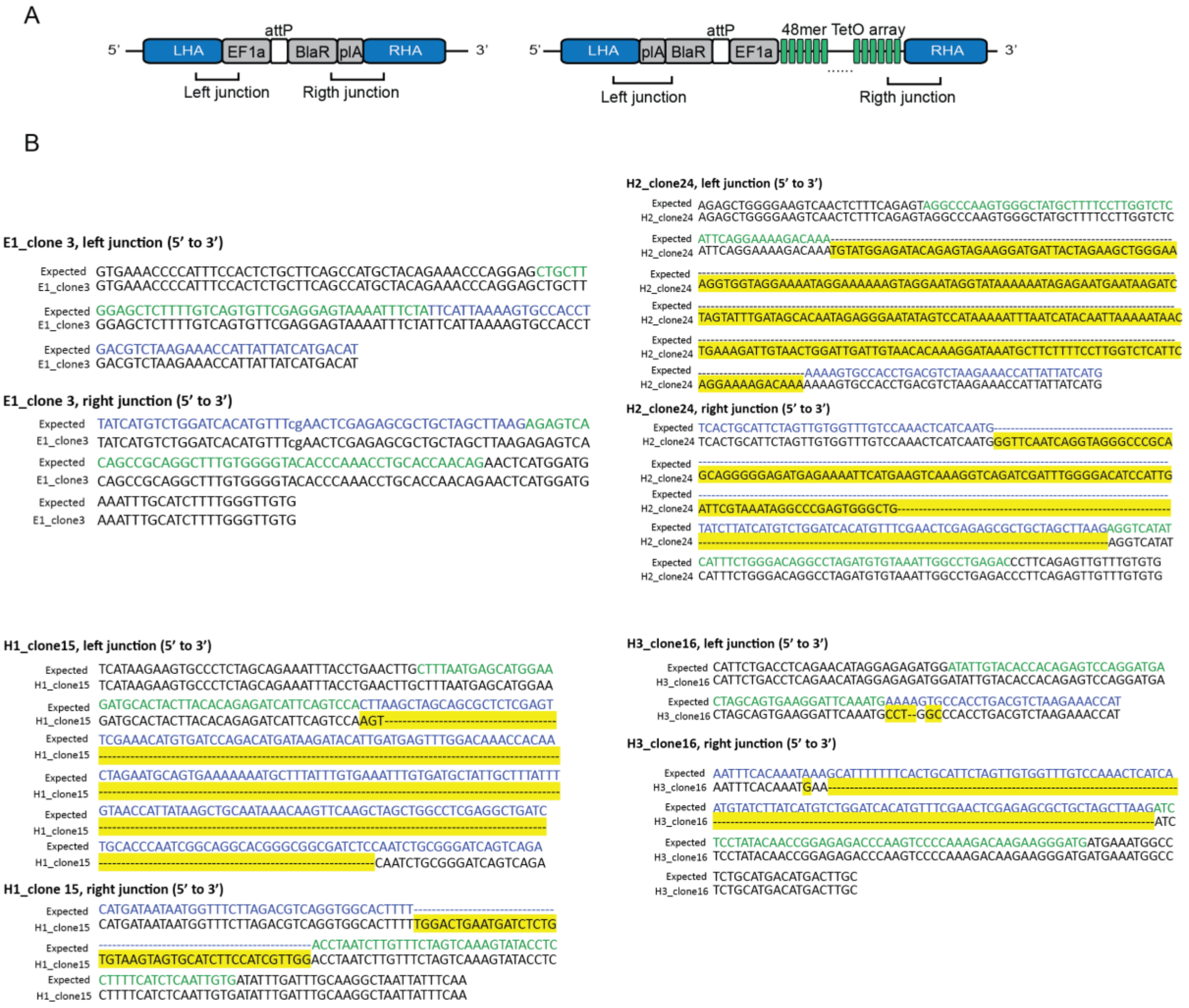

Supplementary Figure 3. Sequencing of knock-in (KI) junctions of selected chassis clones from CRISPR/Cas9-mediated KI. (**A**) Schematic of the KI junctions for E1, H2, H3 (left) and H1 (right). Note: the H1 chassis clone #15 has the donor inserted in the reverse orientation by NHEJ. (**B**) Detailed junction sequencing results of each selected clone with landing pad insertion at E1, H1, H2 or H3. Expected sequence was compiled assuming homology-directed recombination was adopted for donor insertion. Sequences highlighted in yellow were small insertions and deletions (indels).

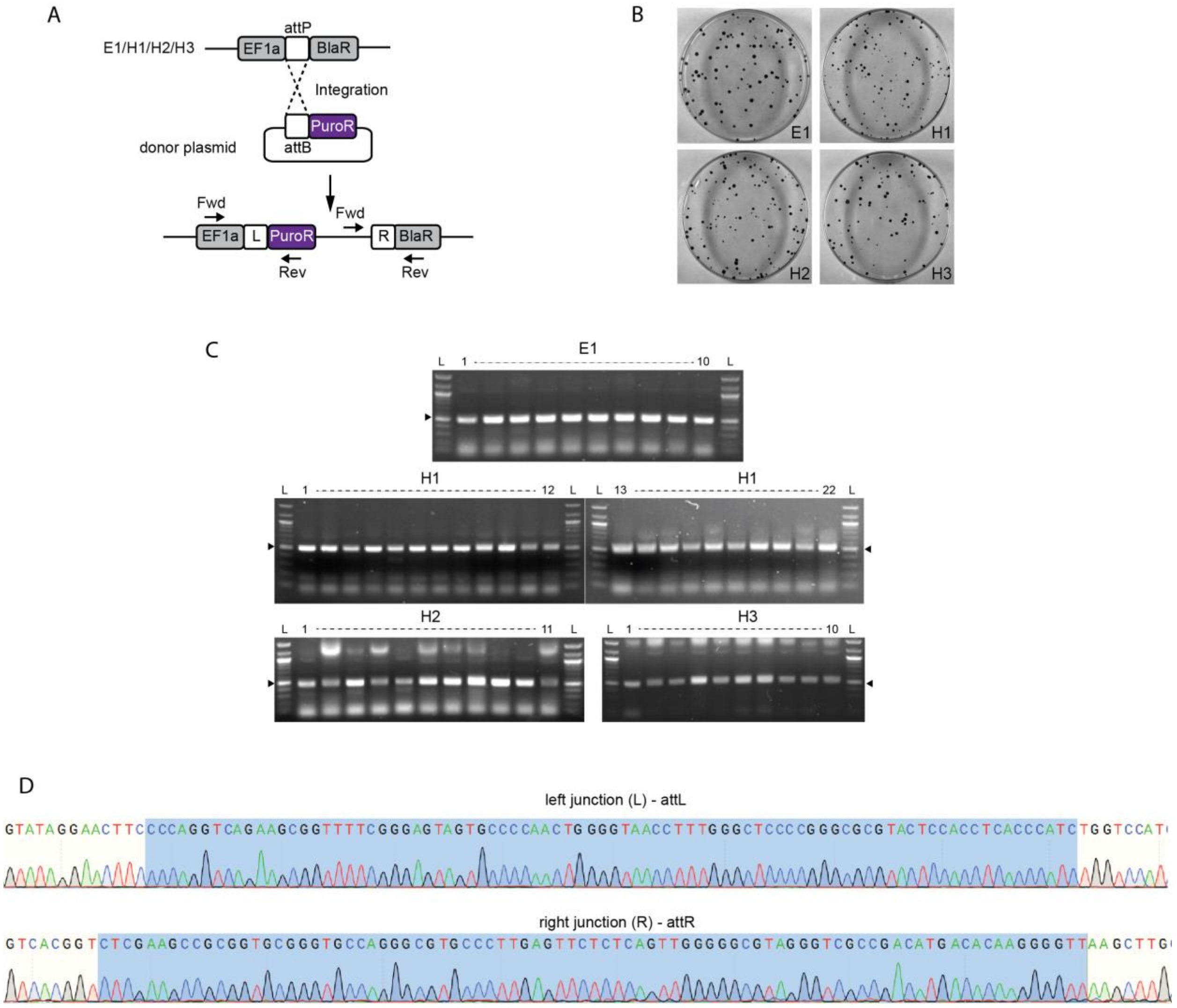

Supplementary Figure 4. Evaluation of integrase activity at four loci. (**A**) Schematic of donor plasmid and genotyping primers (Supplementary Table 4) used to screen for on-target integration. (**B**) Colony formation assay in 100 mm culture plates where colonies surviving puromycin selection were stained by the crystal violet solution. (**C**) PCR results of isolated clones derived from each chassis cell line using primers shown in (A). (**D**) Representative sequencing trace files of the integration junctions from selected clones. For each junction (attL or attR), we sequenced ten clones in total, including one clone for E1 and three clones each for H1-H3.

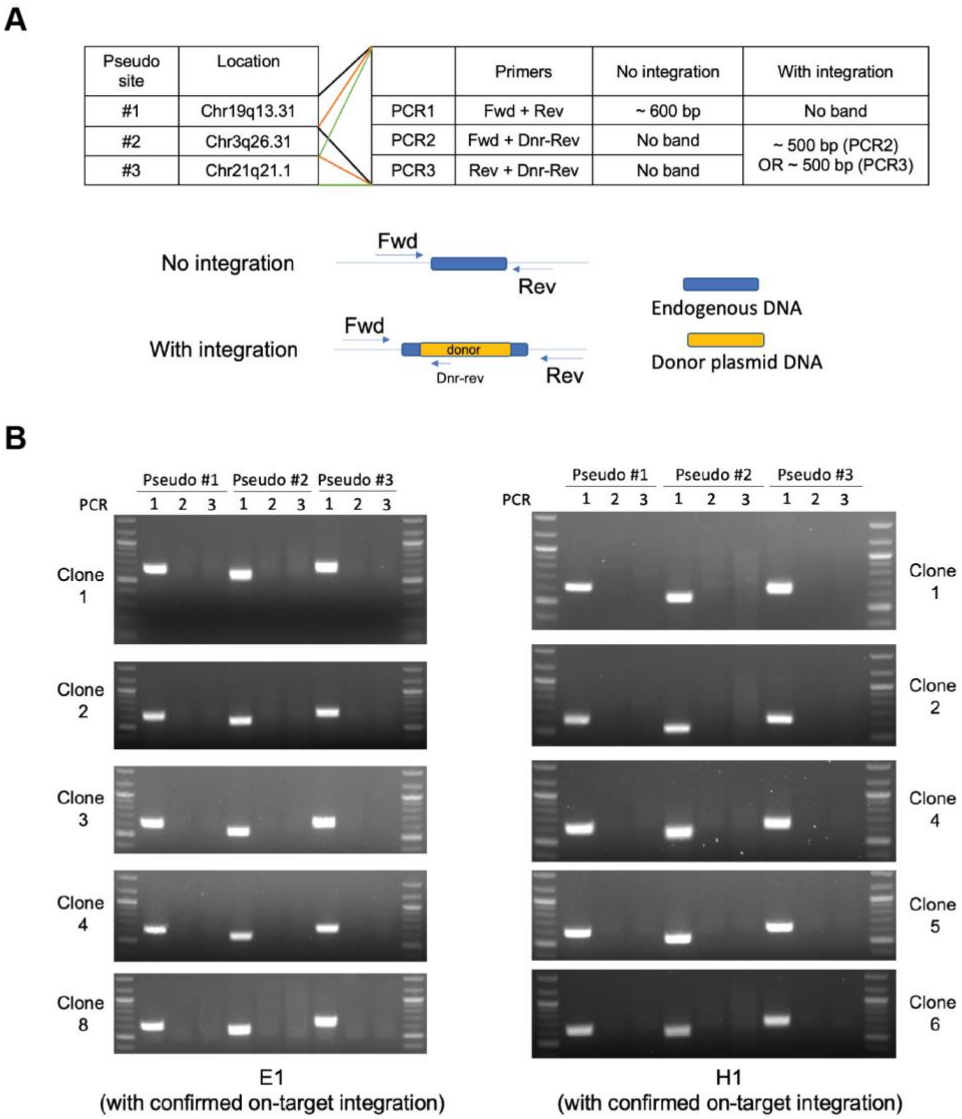

Supplementary Figure 5. A PCR-based assay to assess potential off-target integration at endogenous pseudo sites. **(A)** Left table, top three PhiC31 integrase pseudo sites previously identified from human genome[1]. Right table: three PCRs designed for each pseudo site to assess potential off-target integration, with expected PCR results shown for each scenario (no integration or with integration). Bottom, schematic showing the annealing positions of designed PCR primers. Sequences of primers described here are listed in Supplementary Table 5. **(B)** PCR results of 10 colonies with confirmed on-target integration. All PCR results suggest no donor plasmid insertion at the corresponding pseudo site. Ladder: NEB 100 bp ladder.

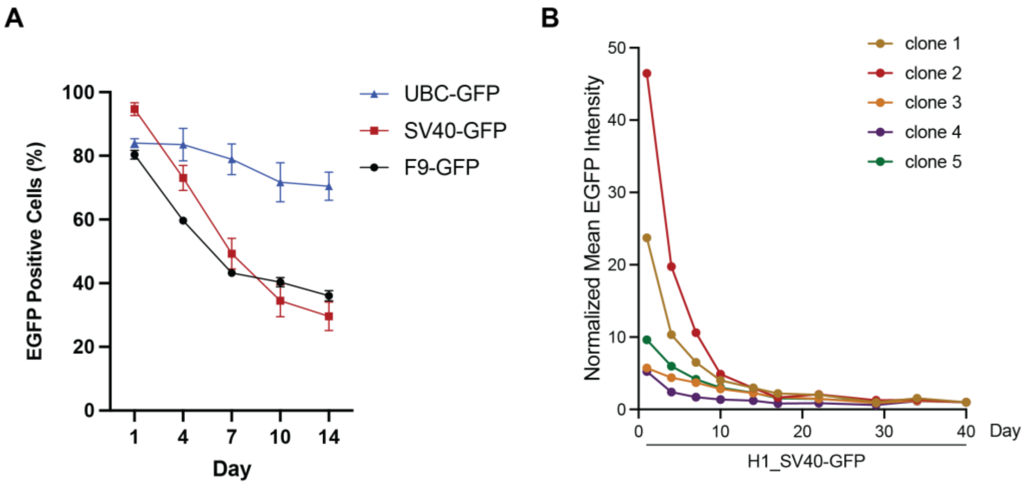

Supplementary Figure 6. (**A**) EGFP^{+} population percentage over time where the indicated reporter cassette was inserted at H1 *via* SHIELD. N = 3. Error bars represent means ± SD. (**B**) EGFP silencing profiles (shown as population mean EGFP intensity over time) of five randomly selected clones with SV40-GFP inserted at H1. For each clone, the EGFP intensity was normalized based on its value on day 40.

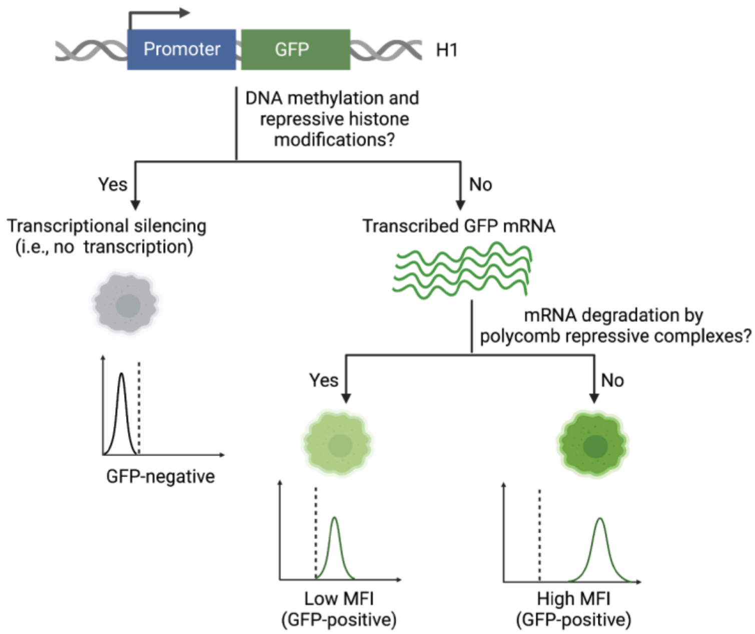

Supplementary Figure 7. Proposed model to explain the emergence of a second $EGFP^+$ population with low intensity during epigenetic silencing at H1. Briefly, transgene silencing at the transcription level (i.e., repressive modification on DNA) results in the $EGFP^-$ population (i.e., complete shutoff, grey cells). For cells where transcription remains active (i.e., $EGFP^+$ population), the transcribed *EGFP* mRNA could be subject to degradation mediated by the polycomb repressive complexes, resulting in the emergence of an $EGFP^+$ population but with lower EGFP intensity.

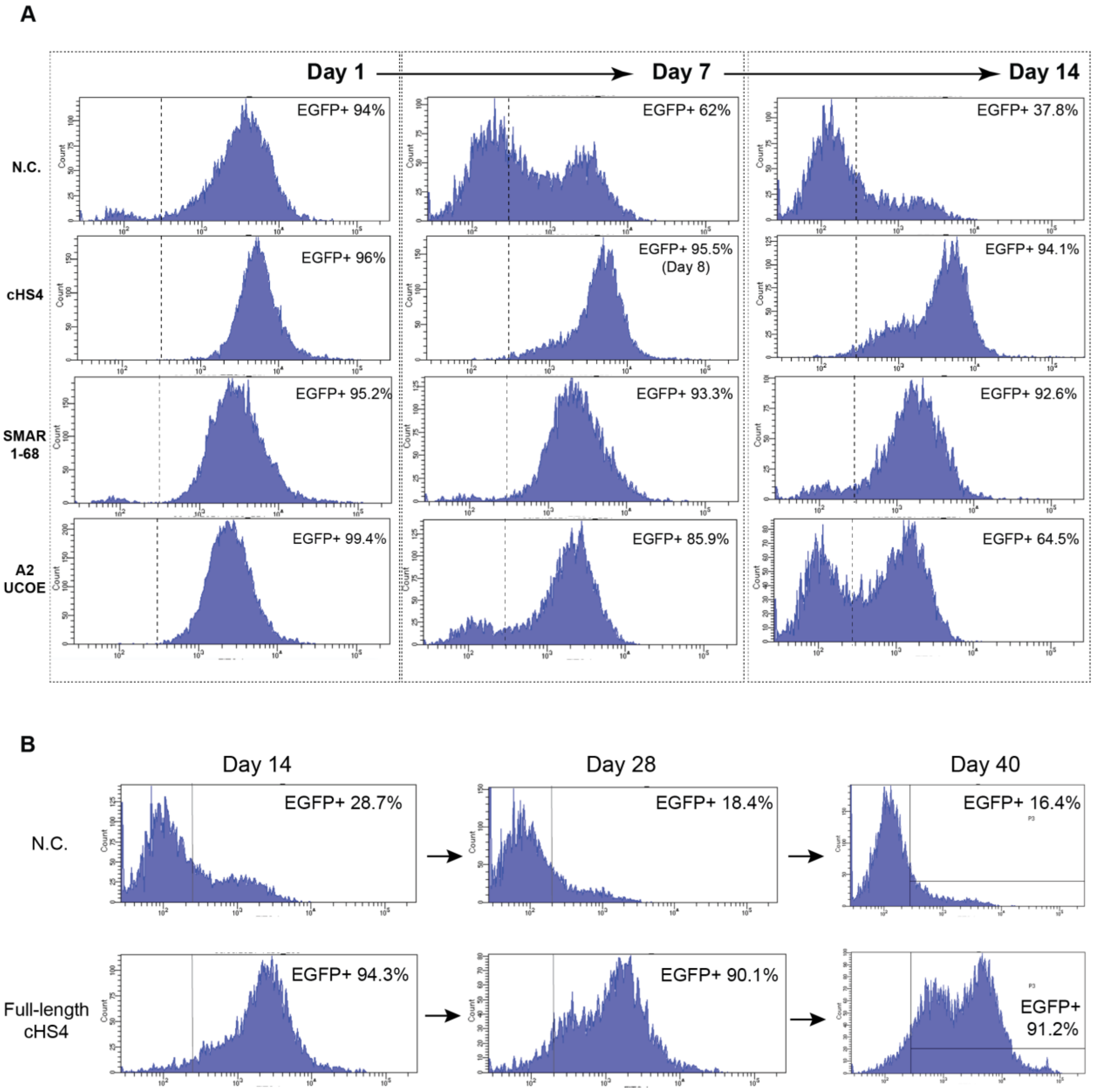

Supplementary Figure 8. (A) Flow cytometry histograms of different populations analyzed on day 1, day 7 and day 14 after puromycin removal. X-axis: FITC (EGFP) signal. Y-axis: counts. Dashed line in each histogram represents the boundary between $EGFP^+$ signal and autofluorescence ($EGFP^-$) as determined by wild-type HCT116 cells. N.C.: negative control, i.e., reporter without any flanking elements. For data shown here, the reporter plasmids were integrated at H1 for a direct comparison of three elements (full-length cHS4, S/MAR 1-68, A2UCOE) under the same chromosome context. **(B)** Flow cytometry histograms of two populations analyzed on day 14, day 28 and day 40 after puromycin removal. N.C.: negative control.

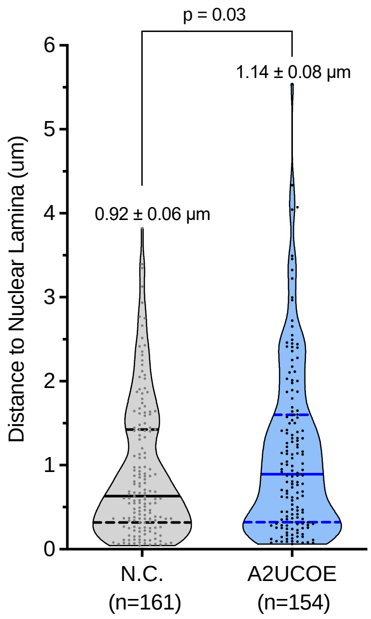

Supplementary Figure 9. Distance distribution of H1 to the nuclear lamina when the reporter plasmid without any flanking element (N.C. = negative control) or with flanking A2UCOE was integrated at H1. Images were collected after 10 days following puromycin removal. Solid line: Median. Dashed line: quartile. Values above each plot: Mean ± SEM (standard error of means). P value was calculated by two-tailed unpaired t-test.

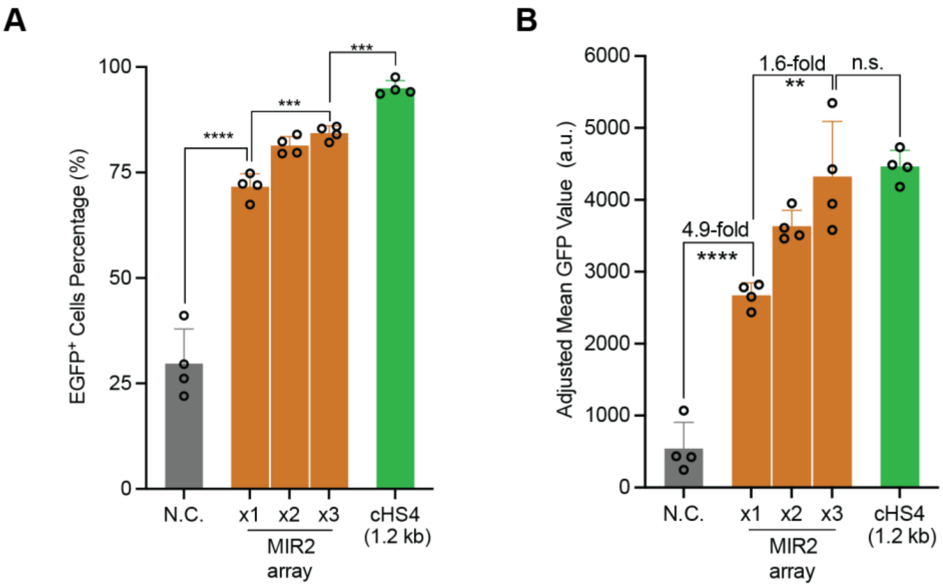

Supplementary Figure 10. Barrier activity of synthetic DNA elements consisting of 1-mer, 2-mer or 3-mer MIR2 arrays. Reporter plasmids (designed as in Figure 4A) carrying the SV40-EGFP reporter gene flanked by either one-copy (x1), two-copy (x2) or three-copy (x3) of MIR2 elements were integrated at H1 via SHIELD. EGFP expression on day 14 was analyzed by flow cytometry and shown in $EGFP^+$ percentage **(A)** or population MFI **(B)**. Error bars represent means ± SD. P value was calculated by two-tailed unpaired t-test, **: p<0.01, ***: p < 0.001, ****: p < 0.0001. n.s. = not significant. N = 4 biological replicates.

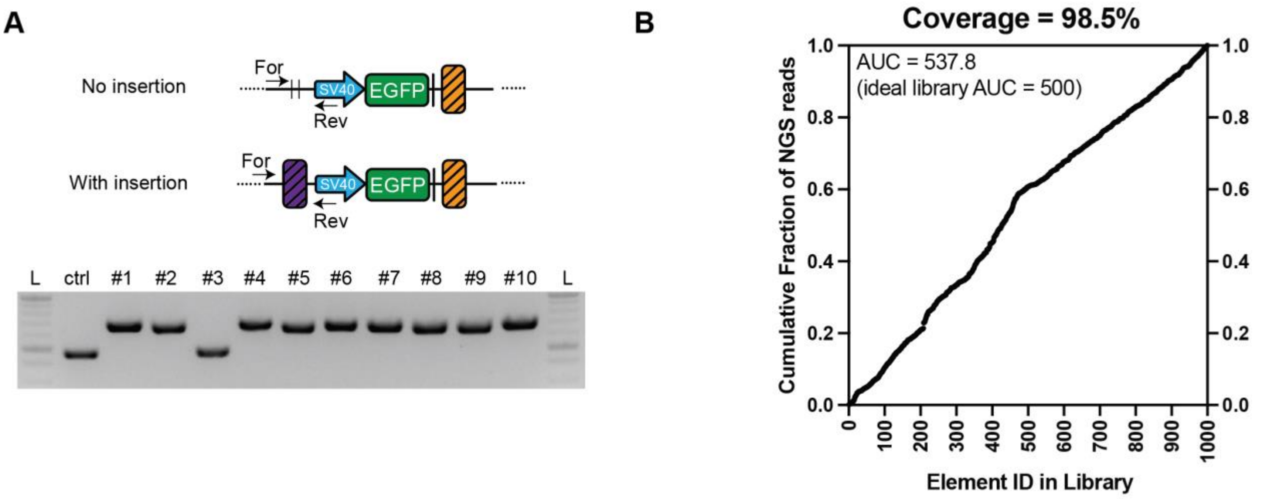

Supplementary Figure 11. (A) Colony PCR-based assay to evaluate the cloning efficiency of library for high-throughput screening. With correct insertion by Golden Gate assembly, PCRs with primers shown would result in amplicons of ~700 bp compared to reporter plasmids with no insertion (expected amplicon ~450 bp). Ctrl: backbone reporter with no insertion. #1 - #10: 10 colonies randomly selected from LB plates. L: NEB 100 bp DNA ladder. Note: plasmid from colony #3 had 34 bp insertion as revealed by Sanger sequencing. However, this 34bp insert could not be mapped to any sequences in the library. **(B)** Cumulative fraction distribution curve of library elements from plasmid maxiprep as determined by NGS. NGS determined a 98.5% coverage of the library, with elements having a relatively equal distribution. AUC: area under the curve. For an ideal library (N=1000) with perfect uniformity, AUC = 500.

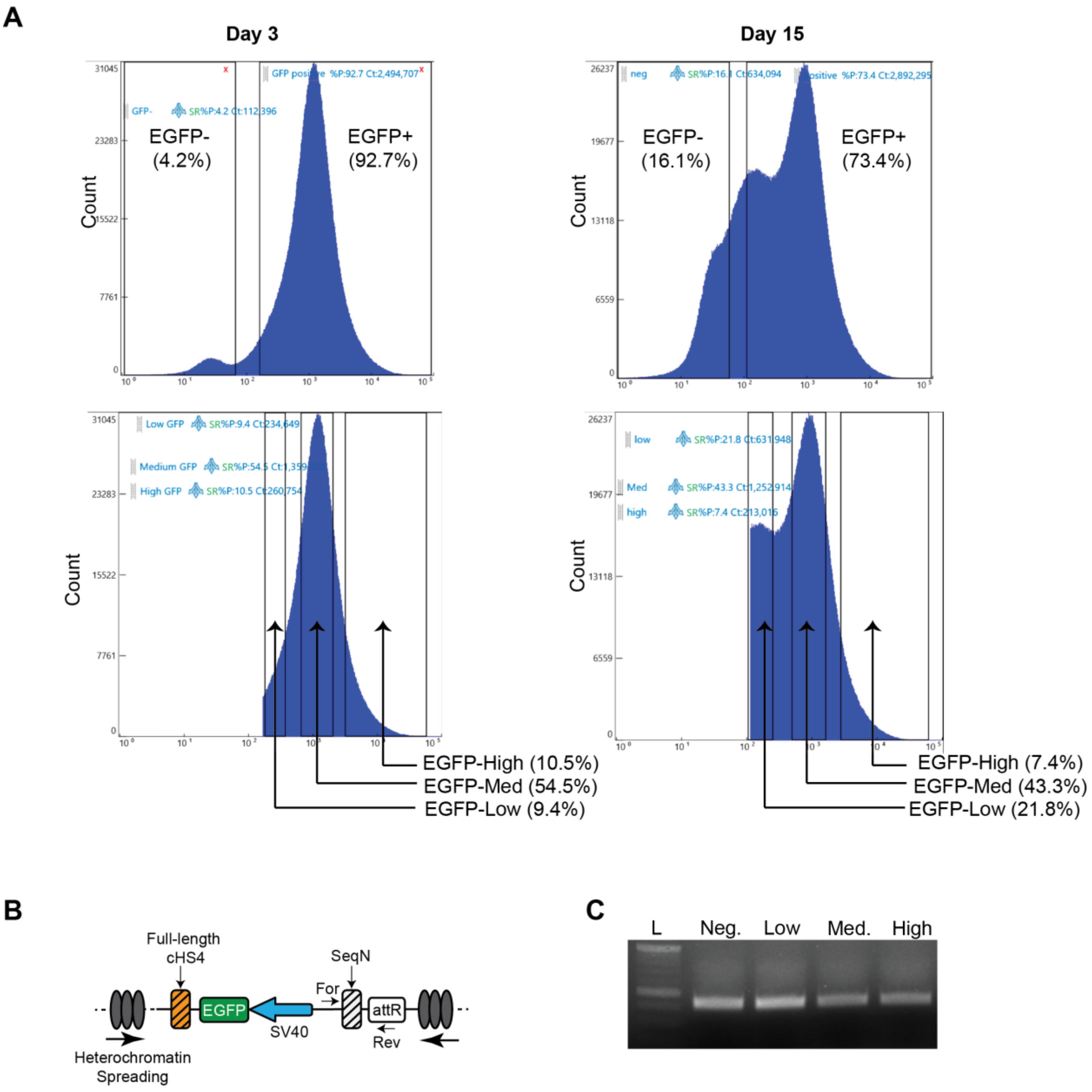

Supplementary Figure 12. Representative images of FACS gating and PCR amplification of library element with integration-specific primers. (**A**) FACS gating for Negative, Low, Medium and High EGFP-expressing populations on day 3 and day 15 following puromycin removal. (**B**) Forward (For) and reverse (Rev) primers designed to amplify library sequences from cells with on-target integration. Primer sequences can be found in Supplementary Table 11. (**C**) Representative gel images of PCR products using primers shown in **(B)** with genomic DNA isolated from the corresponding population. Expected band ~ 430 bp. L: NEB 100 bp ladder.

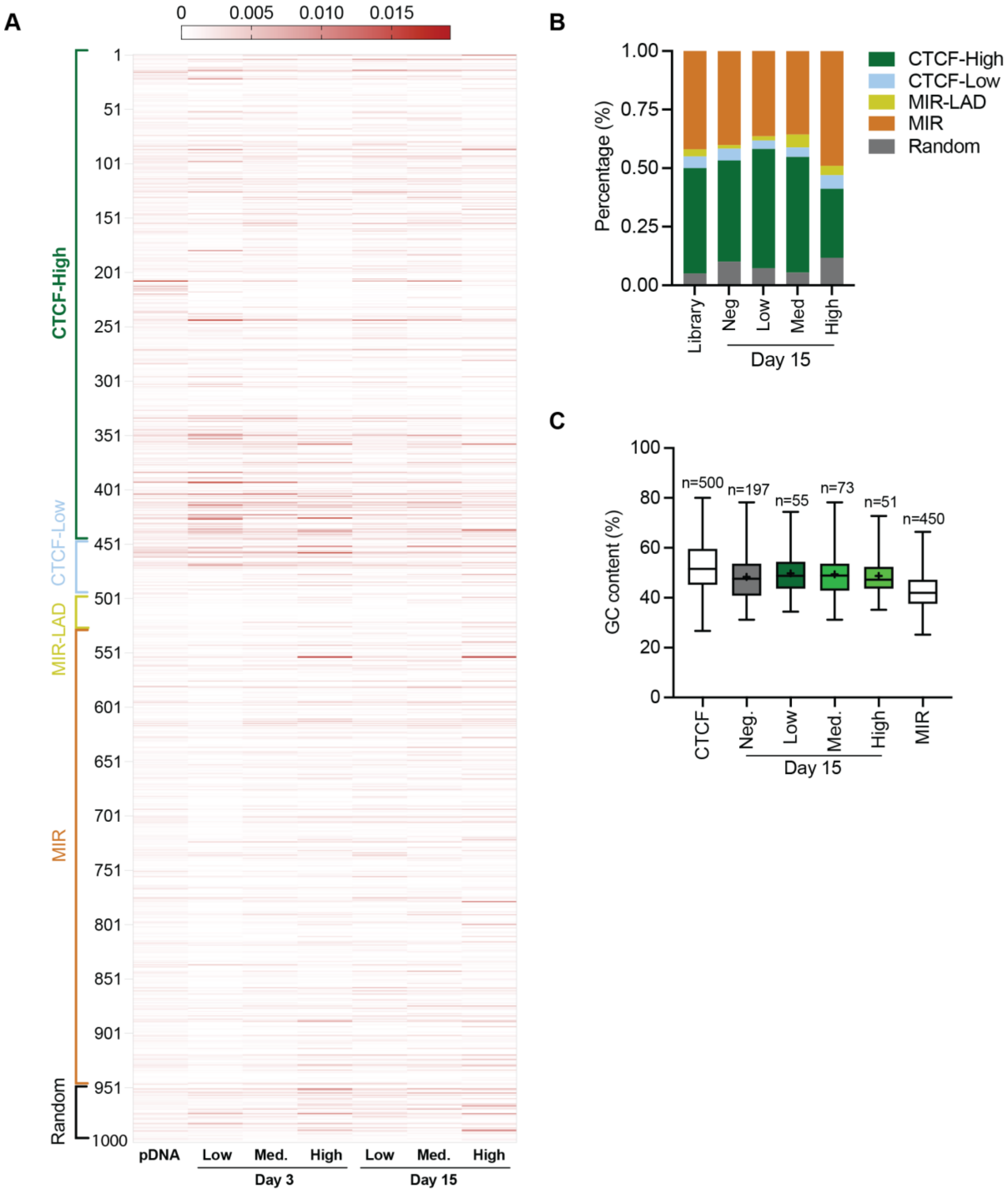

Supplementary Figure 13. **(A)** Heatmap showing the abundance of each element in seven populations, including plasmid DNA library (pDNA), EGFP-Low/Medium/High (day 3) and EGFP-Low/Medium/High (day 15). **(B)** Composition of library elements and elements significantly enriched in four populations sorted on day 15. **(C)** GC content of elements in each group or sorted populations. CTCF: 500 library elements including CTCF-high and CTCF-low affinity binding sites. MIR: 450 library MIR elements.

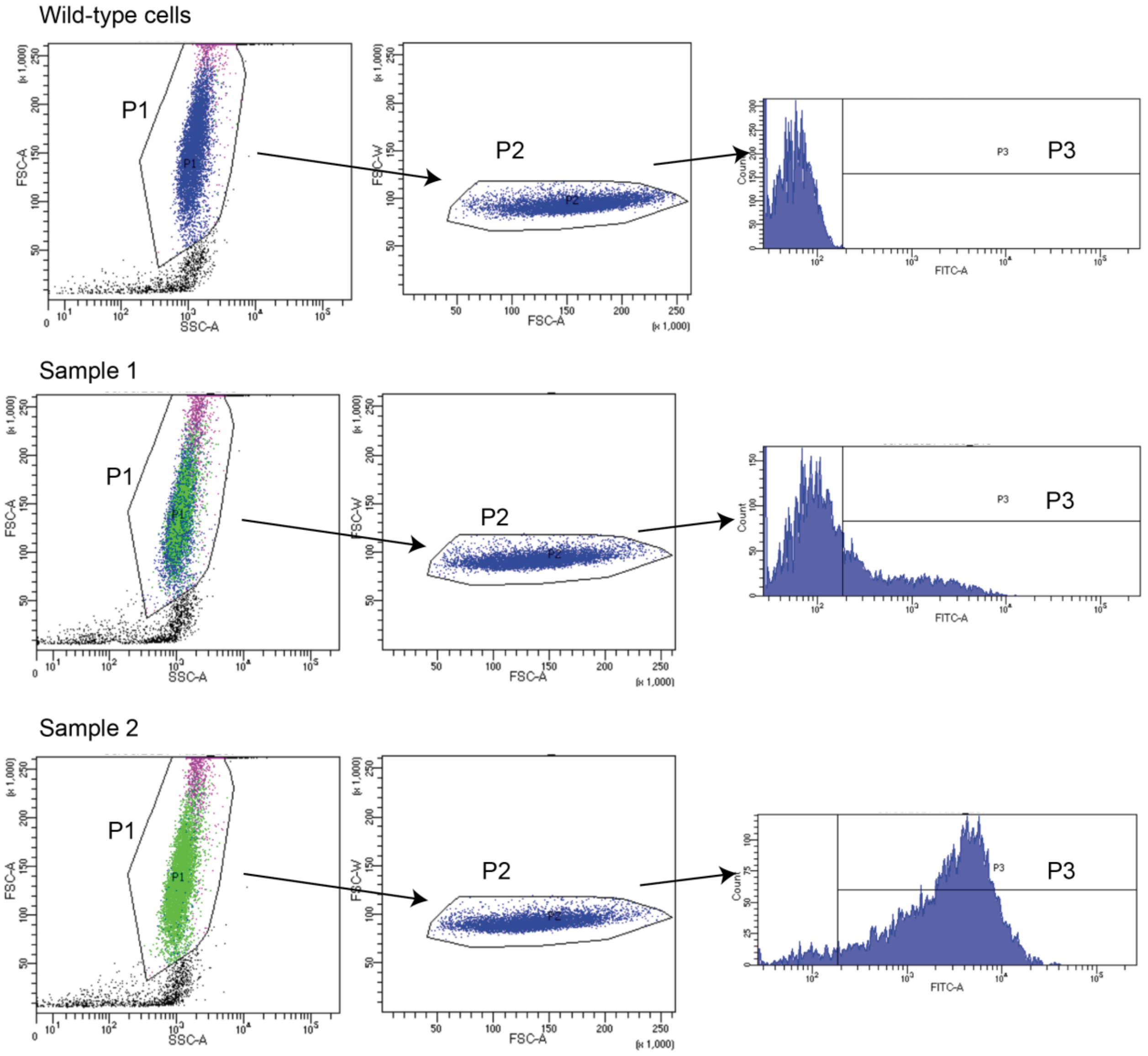

Supplementary Figure 14. Gating strategies for sample analysis by flow cytometry. Shown here are three representative samples analyzed with the same gating set P1, P2 and P3. P1: gating for the main live population of HCT116 cells. P2: daughter gate of P1, gating for the main singlets population. P3: daughter gate of P2, gating for the $EGFP^{+}$ population. Each gated population is also color coded in the FSC-A vs SSC-A plot (P1, purple; P2, blue; P3: green). Note in the FSC-W vs FSC-A plot only population in the P2 gate is shown. Sample 1: negative control (no flanking elements) on day 17. Sample 2: reporter flanked with full-length cHS4 element on day 17. The population MFI and percentage of EGFP+ population reported in the corresponding figures were obtained from P2 and P3, respectively.

Supplementary Table 1. Chromosome coordinates, sgRNAs and genotyping PCR primers used for four selected loci

Target	Chromosome coordinate (hg19)	sgRNA (+ strand, 5' to 3', PAM underlined)	Genotyping primers (5' to 3')
E1	chr6:31,813,898-31,813,920	CCCATTGGCCAGAGTCACAGCCG	Fwd: GCTTCAGCCATGCTACAGAAAC<br>Rev: ACATTACGTACCACTAGTTGTTGGT
H1	chr2:23,061,099-23,061,121	GATCATTCAGTCCAAGTCGTTGG	Fwd: TTTAAATTCCATAATGAGGCCTTGCAC<br>Rev: CATTGACACAGGTATGACTTGTTAGTG
H2	chr7:114,357,720- 114,357,742	CAGGAAAAGACAAATGTCGCTGG	Fwd: GGGTCATGGAGTTAGTGCCAG<br>Rev: CTAAAGACCTCCTGTGTGGCAC
H3	chr11:5,228,205-5,228,227	CCTGGCGAAATCTCCTATACAAC	Fwd: TGCCTACTTCATCCCCAACAG<br>Rev: CCAGCCATCCCTCATTTGG

Supplementary Table 2. Sequence of the landing pad and homology arm sequences for each target locus

Landing pad cassette: FRT-EFS-FRT-attP-BlaR-SV40pIA

5'-aaaagtgccacctgacgtctaagaaaccattattatcatgacattaacctataaaaataggcgtatc
acgaggccctttcgtcttcaagaattcgccctaggggatgaagttcctattctctagaaagtataggaa
cttctaggtcttgaaaggagtgggaattggctccggtgcccgtcagtgggcagagcgcacatcgcccac
agtccccgagaagttggggggaggggtcggcaattgatccggtgcctagagaaggtggcgcggggtaaa
ctgggaaagtgatgtcgtgtactggctccgcctttttcccgagggtgggggagaaccgtatataagtgc
agtagtcgccgtgaacgttctttttcgcaacgggtttgccgccagaacacagggaagttcctattctct
agaaagtataggaacttcccaggtcagaagcggttttcgggagtagtgccccaactggggtaaccttt
gagttctctcagttgggggcgtagggtcgccgacatgacacaaggggttaagcttgccaccatggccaa
gcctttgtctcaagaagaatccaccctcattgaaagagcaacggctacaatcaacagcatccccatctc
tgaagactacagcgtcgccagcgcagctctctctagcgacggccgcatcttcactggtgtcaatgtata
tcattttactggggaccttgcgcagaactcgtggtgctgggcactgctgctgctgcggcagctggcaa
cctgacttgtatcgtcgcgatcggaaatgagaacaggggcatcttgagcccctgcggacggtgccgaca
ggttcttctcgatctgcatcctgggatcaaagccatagtgaaggacagtgatggacagccgacggcagt
tgggattcgtgaattgctgccctctggttatgtgtgggagggctaaccctttatacgcaagggagtagat
gccgaccgaacaagagctgatttcgagaacgcctcagccagcaactcgcgcgagcctagcaaggcaaat
gcgagagaacggccttacgcttggtggcacagttctcgtccacagttcgctaagctcgctcggctgggt
cgcgggagggccggtcgcagtgattcaggcccttctggattgtgttggtccccagggcacgattgtcat
gcccacgcactcgggtgatctgactgatcccgcagattggagatcgccgcccgtgcctgccgattgggt
gcagatcagcctcgaggccagctagcttgaacttgtttattgcagcttataatggttacaaataaagca
atagcatcacaaatttcacaaataaagcatttttttcactgcattctagttgtggtttgtccaaactca
tcaatgtatcttatcatgtctggatcacatgtttcgaactcgagagcgctgctagcttaag-3'

	Left homology arm (5'-3')	Right homology arm (5'-3')
E1	ctgcttggagctcttttgtcagtgttcgagg agtaaaatttctattcatt	agagtcacagccgcaggctttgtggggtaca cccaaacctgcaccaacag
H1	ctttaatgagcatggaagatgcactacttac acagagatcattcagtcca	acctaatcttgtttctagtcaaagtatacct ccttttcatctcaattgtg
H2	aggcccaagtgggctatgcttttccttggtc tcattcaggaaaagacaaa	aggtcatatcatttctgggacaggcctagat gtgtaaattggcctgagac
H3	atattgtacaccacagagtccaggatgacta gcagtgaaggattcaaatg	atctcctatacaaccggagagacccaagtcc ccaaagacaagaagggatg

*For target H1, the landing pad cassette included an extra ~ 1.5 kb DNA consisting of an optimized 48mer TetO repeat array upstream of the "FRT-EFS-FRT" for imaging purpose[2].

Supplementary Table 3. Sources of epigenetic information in HCT116 cell line

Name	Source
RNA-seq	ENCODE/Caltech (wgEncodeEH001425)
LMNB1 DamID	4D Nucleome, Van Steensel lab (HCT116_LMNB1-20kb-combined_ts20180203)[3]
H3K4me1	ENCODE/SYDH (wgEncodeEH002874)
H3K27ac	ENCODE/SYDH (wgEncodeEH002873)
DNaseI HS	ENCODE Open Chromatin by DNaseI HS and FAIRE (wgEncodeEH001162)
H3K9me3	ENCODE (ENCSR000FCP)
H3K27me3	ENCODE (ENCSR810BDB)
Methyl-RRBS	ENCODE/HudsonAlpha (wgEncodeEH001356)
CTCF ChIP-Seq	ENCODE/UW (wgEncodeEH002055)

Supplementary Table 4. Genotyping PCR primers for on-target integration screening

	Fwd (5'-3')	Rev (5'-3')
Left junction (attL)	GCAATTGATCCGGTGCCTAG	CGCGCGTGAGGAAGAGTTCTT
Right junction (attR)	CCGAAAAGTGCCACCTGAC	CAGAGATGGGGATGCTGTTG

Supplementary Table 5. Primers used to assess potential off-target integration at endogenous pseudo sites

	Fwd (5'-3')	Rev (5'-3')
Pseudo #1	GGGTAGCAGATGGAACACTTAGC	ATAGTCCCAGCGACAGTGAGC
Pseudo #2	AGGGGCCCTATAAACCTTGCTG	GGAAGGGGTCGAGAGTGAAGAAA
Pseudo #3	TGCCAAAACCCCCACATTGG	CACAAATGTCTTGCCAGTAAGTCCA
Dnr-Rev (5'-3')	CGCGCGTGAGGAAGAGTTCTT	

Supplementary Table 6. Sequences of the F9, SV40 and UBC promoter

F9:
5’-acgcgtatagatctggatcccgccatggtatcaacgccatatttctatttacagtagggacctcttc
gttgtgtaggtaccgctgtattcctagggaaatagtagaggcaccttgaactgtctgcatcagccatat
agcccccgctgttcgatttacaaacacaggcacagtactgacaaacccatacacctcctctgaaatacc
catagttgctagggctgtctccgaactcattacaccctccaaagtcagagctgtaatttcgccatcaag
ggcagcgagggcttctccagataaaatagcttctgccgagagtcccgtaagggtagacacttcagctaa
tccctcgatgaggtctactagaatagtcagtgcggctcccattttgaaaattcacttacttgatcagct
tcagaagatggcggagggcctccaacacagtaattttcctcccgactcttaaaatagaaaatgtcaagt
cagttaagcaggaagtgactaactgaccgcagctggccgtgcgacatcctcttttaattagttgctagg
caactgccctccagagggcagtgtggttttgcaagaggaagcaaaaagcctctccacccaggcctggaa
tgtttccacccaatcattactatgacaacagctgtttttttttagtattaagcagaggccgggggcccct
ggcctccgcttactctggagaaaaagaagagaggcattgtagaggcttccagaggcaacttgtcaaaac
aggactggcgccttggaggcgctgtggggccacccaaattgatataattaagccccaaccgcctcttcc
cgcctccagatctccattcgccattcaggctgcgcaactgttgggaagggcgatcgtctagag-3’

SV40:
5’-gtgtgtcagttagggtgtggaaagtccccaggctccccagcaggcagaagtatgcaaagcatgcatc
tcaattagtcagcaaccaggtgtggaaagtccccaggctccccagcaggcagaagtatgcaaagcatgc
atctcaattagtcagcaaccatagtcccgcccctaactccgcccatcccgcccctaactccgcccagtt
ccgcccattctccgccccatggctgactaattttttttatttatgcagaggccgaggccgcctctgcct
ctgagctattccagaagtagtgaggaggcttttttggaggcctaggcttttgcaaa-3’

UBC:
5’-ggcctccgcgccgggttttggcgcctcccgcgggcgcccccctcctcacggcgagcgctgccacgtc
agacgaagggcgcaggagcgtcctgatccttccgcccggacgctcaggacagcggcccgctgctcataa
gactcggccttagaaccccagtatcagcagaaggacattttaggacgggacttgggtgactctagggca
ctggttttctttccagagagcggaacaggcgaggaaaagtagtcccttctcggcgattctgcggaggga
tctccgtggggcggtgaacgccgatgattatataaggacgcgccgggtgtggcacagctagttccgtcg
cagccgggatttgggtcgcggttcttgtttgtggatcgctgtgatcgtcacttggtgagtagcgggctg
ctgggctggccggggctttcgtggccgccgggccgctcggtgggacggaagcgtgtggagagaccgcca
agggctgtagtctgggtccgcgagcaaggttgccctgaactgggggttggggggagcgcagcaaaatgg
cggctgttcccgagtcttgaatggaagacgcttgtgaggcgggctgtgaggtcgttgaaacaaggtggg
gggcatggtgggcggcaagaacccaaggtcttgaggccttcgctaatgcgggaaagctcttattcgggt
gagatgggctggggcaccatctggggaccctgacgtgaagtttgtcactgactggagaactcggtttgt
cgtctgttgcgggggcggcagttatgcggtgccgttgggcagtgcacccgtacctttgggagcgcgcgc
cctcgtcgtgtcgtgacgtcacccgttctgttggcttataatgcagggtggggccacctgccggtaggt
gtgcggtaggcttttctccgtcgcaggacgcagggttcgggcctagggtaggctctcctgaatcgacag
gcgccggacctctggtgaggggagggataagtgaggcgtcagtttctttggtcggttttatgtacctat
cttcttaagtagctgaagctccggttttgaactatgcgctcggggttggcgagtgtgttttgtgaagtt
ttttaggcaccttttgaaatgtaatcatttgggtcaatatgtaattttcagtgttagactagtaaattg
tccgctaaattctggccgtttttggctttttttgttagac-3’

Supplementary Table 7. Sequences of cHS4, A2-UCOE and S/MAR-68

cHS4:<br>5’-gctagagggacagccccccccaaagccccagggatgtaattacgtccctcccccgctagggggca<br>gcagcgagccgcccggggctccgctccggtccggcgctccccccgcatccccgagccggcagcgtgcgg<br>ggacagcccgggcacggggaaggtggcacgggatcgctttcctctgaacgcttctcgctgctctttgag<br>cctgcagacacctgggggatacggggaaaagctttaggctgaaagagagatttagaatgacagaatc<br>atagaacggcctgggttgcaaaggagcacagtgctcatccagatccaacccctgctatgtgcagggtc<br>atcaaccagcagcccaggctgcccagagccacatccagcctggccttgaatgcctgcagggatggggca<br>tccacagcctccttgggcaacctgttcagtgcgtcaccaccctctgggggaaaaactgcctcctcatat<br>ccaacccaaacctcccctgtctcagtgtaaagccattccccctttgtcctatcaaggggggagtttgctgt<br>gacattgttggtctggggtgacacatgtttgccaattcagtgcatcacggagaggcagatcttggggat<br>aaggaagtgcaggacagcatggacgtgggacatgcaggtgttgagggctctgggacactctccaagtca<br>cagcgttcagaacagccttaaggataagaagataggatagaaggacaaagagcaagttaaaacccagca<br>tggagaggagcacaaaaaggccacagacactgctggtccctgtgtctgagcctgcatgtttgatggtgt<br>ctggatgcaagcagaaggggtggaagagcttgcctggagagatacagctgggtcagtaggactgggaca<br>ggcagctggagaattgccatgtagatgttcatacaatcgtcaaatcatgaaggctggaaaagccctcca<br>agatccccaagaccaaccccaacccacccaccgtgcccactggccatgtccctcagtgccacatcccca<br>cagttcttcatcacctccagggacggtgaccccccacctccgtgggcagctgtgccactgcagcaccg<br>ctctttggagaaggtaaatcttgctaaatccagcccgaccctcccctggcacaacgtaaggccattatc<br>tctcatccaactccaggacggagtcagtgaggatggggctctag-3’<br>*Underlined is the 300bp core sequence.
A2UCOE:<br>5’-cgcagcctcgctcacgaggacctgctgcccgccgaaacgctcgccgaggagacgccgtggcccccga<br>agcagcgtgctttagaaagggaataagaattcccgcctccgcgccccactttcaccccagcggggcagc<br>gtccgccatgtgaaagctccccatcccccacccccagtgaagggaaatggcgccgggaggctgagggtg<br>gggaagctgtttgtacgctcaggcctccgctcaagaccccgttcataaaccttaagccccactgctact<br>gaattggtccgatttcctgcctctctcccacggaggcggctggccgacttccactgaggcgccaacggc<br>ctcgccatgccctttttcaataactcattgatttcaaacccgttacctccatcgcggactcagtcgcttc<br>agcccgatttcccgcagccgagcgagatgagagagatctccgcggacgaacacgaaccggactcgtcct<br>ggcgctgtagtgagaactgccgctgctcgagaaacaactctgcgaggagcacctccgcacgggacccgg<br>cgctgctgctactgccgctagagccgctgccgccgcttttctagaaccttccccccactaacgcgtct<br>tccgctacgtcaggccgtcgcgtaaacgccctatccgccgccaatggcgggaaggctctacgccccacc<br>ttacgccaaatgcgtactcctcccacccttgcggccagagacagtacccgacgttacttccgtaaatgc<br>gctcaatgaattgcggaaggctagagtcctgctagttactacctcttggaatagggtcccgccccctgc<br>cttggcgcaaggcaggtgagaaacggtcgcgcagtttgaaattaacgccgacgggaggggcttaatccg<br>cagcctggagatccagcccctcaacccgggaggtggtccctgcagttacgccaatgataacccccgcc<br>agaaaaatcttagtagccttccctttttgttttccgtgccccaactcggcggattgactcggccccttc<br>cggaaacacccgaatcaacttctagtcaaattattgttcacgccgcaatgacccacccctggcccgcgt<br>ctgtggaactgacccctggtgtacaggagagttcgctgctgaaagtggtcccaaaggggtactagtttt<br>taagctcccaactcccccctcccccagcgtctggaggattccacaccctcgcaccgcagggggcgaggaag<br>tgggcggagtccggttttggcgccagccgctgaggctgccaagcagaaaagccaccgctgaggagactc<br>cggtcactgtcctcgccccgcctccccccttccctccccttggggaccaccgggcgccacgccgcgaacg<br>gtaagtgccgcggtcgtcggcgcctccgccctcccccctagggccccaattcccagcgggcgcggcgcgc<br>ggcccctcccccccgccgggcgcgcgcccgctgccccgcccttcgtggccgcccggcgtgggcggtgcca<br>cccctcccccccggcggccccgcgcgcagctcccggctccctccccccttcggatgtggcttgagctgtag<br>gcgcggagggccggagacgctgcagacccgcgacccggagcagctcggaggcggtgaagtcggtggctt<br>tccttctctctagctctcgctcgctggtggtgcttcagatgccacacgcgtcc-3’
S/MAR 1-68:<br>5’-tctagattataccaacctcataaaataagagcatatataaaagcaaatgctcttatcttgcagatcc

```
ctgaactgaggaggcaagatcagtttggcagttgaagcagctggaatctgcaattcagagaatctaaga
aaagacaaccctgaagagagagacccagaaacctagcaggagtttctccaaacattcaaggctgaggga
taaatgttacatgcacagggtgagcctccagaggcttgtccattagcaactgctacagtttcattatct
cagggatcacagattgtgctacctattgcctaccatctgaaaacagttgcttcctatatttcatccagt
ttaatatttatttaaaccaagaaggttaatctggcaccagctattccgttgtgagtggatgtgaaagta
ccaattccattctgttttactattaactatcctttgccttaatatgtatcagtaggtggcttgttgcta
ggaaatattaaatgaatggcatgtttcataggttgtgtttaaagttgtttttgagttaaatctttctt
taataatactttctgatgtcaaaaacacttagaagtcatggtgttgaacatctatatagggttggatct
aaaatagcttcttaacctttcctaaccactgtttttgtttgtttgtttttaactaagcatccagtttgg
gaaattctgaattaggggaatcataaaaggtttcattttagctgggccacataaggaaagtaagatatc
aaattgtaaaaatcgttaagaacttctatcccatctgaagtgtgggttaggtgcctcttctctgtgctc
ccttaacatcctattttatctgtatatatatatattcttccaaatatccatggaaaaaaaatctgatc
ataaaaatatttaggctgggagtggtggctcacgcctgtaatcccagcactttgggaggctgaggtgg
gcggatcatgaggtcaagagatcgagaccatcctgaccaatatggtgaaaccccatctctactaaagat
acaaaactattagctggacgtggtggcacgtgcctgtagtcccagctactcgggaggctgaggcaggag
aacggcttgaacccaggaggtggaggttgcagtgagctgagatcgcgccactgcactccagcctgggcg
acagagcgagactctgtctcaaaaaaaaaatatatatatatatatatacacatatatatataaaata
tatatatatacacacatatatatataaaatatatatatatacacacatatatataaaatatatatatat
acacacatatatataaaatatatatatacacacatatatataaaatatatatatacacacatatatata
aaatatatatatacacacatatatataaaatatatatatacacacatatatataaaatatatatataca
cacatatatataaaatatatatatacacacatatatataaaatatatatatacacacatatatataaaa
tatatatatacacacatatatatataaaatatatatatacacacatatatataaaatatatatatacacac
atatataaaatatatatatacacacatatataaaatatatatatacacatatatataaaatatatatat
acacatatatataaaatatatatacacacatatatataaaatatatatatacacacatatatataaaat
atatatatacacatatatataaaatatatatatacacatatatataaaatatatatatatacacatata
tataaaatatatatacacacatatatataaagtatatatatacacacatatatataaaatatatatata
cacatatatataaaatatatatatacacatatatataaaatatatatatacacatatatataaaatata
tatatacacatatatataaaaatatatatatatattttttaaaatattccaattgtctcactttgtgga
tgagaaaaagaagtagttagaggtcaagtaacttggcctacatcttttctcaagattgtaaactcctag
tgagcaataaccacatcttcatttctttgtataaaacaagaaagtttagcatgaaaaaggtactcaat
tacaaatgtgttggattgaattgaagacccttggaaggggattttgtacctgaggatctctttcttttg
gccatattgttcaatggacaaaatttagccttcgaaggcaggccgatttgaggttaatactacctttac
cacttgatagctatgtgaccttggccatgtggtttcaacagtctgaacctcatttctctgtgtatgtg
tggtcctccttacaagtttgtgaaaaatgtgaagtccttagccatgatagcccaatataacaggctaaa
tgataataggtttatgttcttttcctttatattctcagataagcactgtccaagtttgaggtgttttga
ggtctcgcctgatttggattgtttgagtttatgctattctttgaattctttgagctgttctgaagcagt
gtatcatgaacaaaaacatccccagttcagtccaaaccccctggttacatatcattcttatgccatgtta
taaccagtttgagagtgttccctctgttattgcatttaagtttcagcctcacacagaaattcagcagcc
aatttctaagccctaagcataaaatctggggtgggggggggggatggcctgaagagcagcattatgaat
agcaccattataattaatgatctctcaggaagatttacaatcacaggtagcagataaaacaaatagtac
tgcttctgcacttcccctccttttattcgctatgaaattttatgggaaatcagtccagtgaaaaatgta
agctcttaatctttcccagaaatcctacctcatttgatgaatactttgagggaatgaattagagcattt
ttttcttttatagtctacttcgcatttacgaagtgaggacggtagcttaggctgcctggccaactgatg
agaaggtcagaggcatttttagagacctctgttgtctttcattcatgttcattttccacaaggcaagta
atttccaacaaatcagtgtcttcattagtaataagattattaacaacaataatagtcatagtaactatt
cagtgagagtccattatatatcaggcattctacaaggtactttatatacatctgagtaaacctcacaca
attctacagggaggtatttctatccccatttaacaaataaggaaacgaagtccaagtaaattaacttgc
ccaaggtcacacagatagtacctggcagaacaggaatttaaacctaaatttgtccaactccaaaagcag
ccttctatttgttataaatgctgcctctcattatcacatattttattattaacaacaacaaacatacca
attagcttaagatacaatacaaccagataatcatgatgacaacagtaattgttatactattataataaa
```

atagatgttttgtatgttactataatcttgaatttgaatagaaatttgcatttctgaaagcatgttcct<br>gtcatctaatatgattctgtatctatt-3’

Supplementary Table 8. Chromosome coordinates of A2, A4, E2, MIR1, MIR2, MIR3

	hg19	Reference
A2 (CTCF)	chr19:41650330-41650595	Liu *et al.*[4]
A4 (CTCF)	chr7:39559582-39559824	
E2 (CTCF)	chr13:21498993-21499294	
MIR1	chr1:23683272-23683501	Wang *et al.*[5]
MIR2	chr2:98633063-98633436	
MIR3	chr11:82611902-82612195	

Supplementary Table 9. Sequence feature comparison of A2, A4, E2, MIR1, MIR2 and MIR3

	Name	Size (bp)	CTCF ChIP-Seq	VEZF1 (5'-GGGG-3')	USF (5'-CANNTG-3')	Relative barrier activity by SHIELD
CTCF-high affinity binding sites	A2	272	Very high (181/194)	0	1	★
	A4	243	Very high (180/194)	2	1	★★
	E2	302	Very high (178/194)	0	2	None
MIR retrotransposon sequences	MIR1	230	None	2	2	None (large variation)
	MIR2	374	Low (1/194)	4	3	★★★
	MIR3	294	Medium (68/194)	0	2	None (large variation)
Ctrl element	cHS4	1215	High*	26	6	★★★★★

*For cHS4, its CTCF-binding affinity (High) is not directly comparable to other elements as it is determined in a different organism.

Supplementary Table 10. Primers used to amplify DNA oligos for the construction of reporter plasmid library

Fwd (5'-3')	TATCTACACGCGTCTCAGGCTAAC
Rev (5'-3')	GAGTTACGCTCGTCTCTCCATAAAAC

Supplementary Table 11. Primers used to amplify DNA element from sorted populations with on-target integration

Fwd (5'-3')	<u>TCGTCGGCAGCGTCAGATGTGTATAAGAGACAG</u>ACACATTCCACAGAATTAATTCGCGAC
Rev (5'-3')	<u>GTCTCGTGGGCTCGGAGATGTGTATAAGAGACAG</u>ACGCCCCCAACTGAGAGAAC

*Underlined sequences are overhang adapters for Illumina sequencing.

Supplementary Table 12. Information of top 2 hits from high-throughput screening

	Coordinate (hg19)	Class	GC content	CTCF-binding affinity	VEZF1 binding motif (5'-GGGG-3')	USF binding motif (5'-CANNTG-3')
CTCF268	chr5:64080303-64080552	CTCF-high affinity	36%	High	1	1
MIR801	chr19:47083200-47083449	MIR	49%	No peak detected	5	1

References

1. Chalberg, T. W. *et al.* Integration Specificity of Phage ϕC31 Integrase in the Human Genome. *Journal of Molecular Biology* **357**, 28–48 (2006).
2. Tasan, I. *et al.* CRISPR/Cas9-mediated knock-in of an optimized TetO repeat for live cell imaging of endogenous loci. *Nucleic Acids Research* **46**, e100–e100 (2018).
3. van Schaik, T., Vos, M., Peric-Hupkes, D., HN Celie, P. & van Steensel, B. Cell cycle dynamics of lamina-associated DNA. *EMBO reports* **21**, e50636 (2020).
4. Liu, M. *et al.* Genomic discovery of potent chromatin insulators for human gene therapy. *Nat Biotechnol* **33**, 198–203 (2015).
5. Wang, J. *et al.* MIR retrotransposon sequences provide insulators to the human genome. *Proc Natl Acad Sci USA* **112**, E4428–E4437 (2015).
