# Supplementary material for: SHIELD: a platform for high-throughput screening of barrier-type DNA elements in human cells
Source: Nat Commun. 2023 Sep 12;14:5616. doi: 10.1038/s41467-023-41468-3 (PMC10497619; doi:10.1038/s41467-023-41468-3)
Supplement: Supplementary file 2 — Reporting Summary [file 41467_2023_41468_MOESM2_ESM.pdf]

nature portfolio

Corresponding author(s): Huimin Zhao

Last updated by author(s): Aug 25, 2023

Reporting Summary

Nature Portfolio wishes to improve the reproducibility of the work that we publish. This form provides structure for consistency and transparency in reporting. For further information on Nature Portfolio policies, see our Editorial Policies and the Editorial Policy Checklist.

Statistics

For all statistical analyses, confirm that the following items are present in the figure legend, table legend, main text, or Methods section.

n/a	Confirmed	
☐	☒	The exact sample size (n) for each experimental group/condition, given as a discrete number and unit of measurement
☐	☒	A statement on whether measurements were taken from distinct samples or whether the same sample was measured repeatedly
☐	☒	The statistical test(s) used AND whether they are one- or two-sided<br>*Only common tests should be described solely by name; describe more complex techniques in the Methods section.*
☒	☐	A description of all covariates tested
☒	☐	A description of any assumptions or corrections, such as tests of normality and adjustment for multiple comparisons
☐	☒	A full description of the statistical parameters including central tendency (e.g. means) or other basic estimates (e.g. regression coefficient) AND variation (e.g. standard deviation) or associated estimates of uncertainty (e.g. confidence intervals)
☐	☒	For null hypothesis testing, the test statistic (e.g. F, t, r) with confidence intervals, effect sizes, degrees of freedom and P value noted<br>*Give P values as exact values whenever suitable.*
☒	☐	For Bayesian analysis, information on the choice of priors and Markov chain Monte Carlo settings
☒	☐	For hierarchical and complex designs, identification of the appropriate level for tests and full reporting of outcomes
☐	☒	Estimates of effect sizes (e.g. Cohen's d, Pearson's r), indicating how they were calculated

Our web collection on statistics for biologists contains articles on many of the points above.

Software and code

Policy information about availability of computer code

Data collection	BD FACSDIVA 8.0 (flow cytometry), Bigfoot spectral cell sorter sasquatch software (FACS), Illumina MiSeq system (NGS),
Data analysis	Analyses and plotting of numerical data (e.g., percentage of GFP+ cells, mean fluorescence intensity, etc.) were performed using GraphPad Prism 9. Analyses of NGS data (e.g., trimming, blast alignment, etc.) were performed with open source toolkits including FASTX-Toolkit and BLAST+ 2.7.1. module. Analyses of microscope images were performed using ImageJ.

For manuscripts utilizing custom algorithms or software that are central to the research but not yet described in published literature, software must be made available to editors and reviewers. We strongly encourage code deposition in a community repository (e.g. GitHub). See the Nature Portfolio guidelines for submitting code & software for further information.

Data

Policy information about availability of data

All manuscripts must include a data availability statement. This statement should provide the following information, where applicable:

- Accession codes, unique identifiers, or web links for publicly available datasets
- A description of any restrictions on data availability
- For clinical datasets or third party data, please ensure that the statement adheres to our policy

Data availability statement:
All data generated or analyzed during this study are included in the main text or supplementary information. Plasmids used in this study will be deposited to

Addgene and are available from the corresponding author upon request. Source data are provided with this paper in a separate Excel file. NGS data can be accessed at GEO with the accession number GSE236198.

Research involving human participants, their data, or biological material

Policy information about studies with human participants or human data. See also policy information about sex, gender (identity/presentation), and sexual orientation and race, ethnicity and racism.

Reporting on sex and gender	not applicable
Reporting on race, ethnicity, or other socially relevant groupings	not applicable
Population characteristics	not applicable
Recruitment	not applicable
Ethics oversight	not applicable

Note that full information on the approval of the study protocol must also be provided in the manuscript.

Field-specific reporting

Please select the one below that is the best fit for your research. If you are not sure, read the appropriate sections before making your selection.

☒ Life sciences ☐ Behavioural & social sciences ☐ Ecological, evolutionary & environmental sciences

For a reference copy of the document with all sections, see nature.com/documents/nr-reporting-summary-flat.pdf

Life sciences study design

All studies must disclose on these points even when the disclosure is negative.

Sample size	No particular statistical methods were used to predetermine the sample size. N = 3, 4, or 5 biological replicates as shown in figure legends. The sample size was chosen based on previous relevant studies as was shown to be sufficient for statistical analysis.
Data exclusions	No data point was excluded for analysis, except for one replicate in the barrier activity test of element A2 shown in Figure 4B and 4C (N = 3 for A2 element, N = 4 for all other elements in the same figure). Rationale for exclusion: (1) There were significantly less cells after puromycin selection from this particular replicate, indicating cells were in an unhealthy state and thus not suitable for analysis and (2) This data point was excluded also because it was classified as an outlier by the Grubb's test (www.graphpad.com/quickcalcs/grubbs1/).
Replication	Colony formation assay was performed with five biological replicates (Figure 2A). All flow cytometry experiments were performed in triplicates or quadruplicates. For high-throughput screening, transfected cells after puromycin selection were pooled and plated per plate onto two 100 mm cell culture plates that served as two biological replicates for epigenetic silencing, FACS and NGS analysis (details can be found in the methods section). Results were reliably reproduced at attempts.
Randomization	Not relevant to this study because we performed experiments with the HCT116 cell line with defined genetic background.
Blinding	Not relevant because no group allocation was involved in this study.

Reporting for specific materials, systems and methods

We require information from authors about some types of materials, experimental systems and methods used in many studies. Here, indicate whether each material, system or method listed is relevant to your study. If you are not sure if a list item applies to your research, read the appropriate section before selecting a response.

Materials & experimental systems

n/a	Involved in the study	
☒	☐	Antibodies
☐	☒	Eukaryotic cell lines
☒	☐	Palaeontology and archaeology
☒	☐	Animals and other organisms
☒	☐	Clinical data
☒	☐	Dual use research of concern
☒	☐	Plants

Methods

n/a	Involved in the study	
☒	☐	ChIP-seq
☐	☒	Flow cytometry
☒	☐	MRI-based neuroimaging

Eukaryotic cell lines

Policy information about cell lines and Sex and Gender in Research

Cell line source(s)	HCT116 was obtained from ATCC.
Authentication	HCT116 was authenticated by ATCC (ATCC Cell Line Authentication Service and Sanger Sequencing)
Mycoplasma contamination	We strictly follow ATCC's recommended guidelines and adopt stringent aseptic practice. We did not test cells for mycoplasma contamination after receiving it from ATCC.
Commonly misidentified lines (See ICLAC register)	No commonly misidentified cell lines used in this study.

Flow Cytometry

Plots

Confirm that:

- [x] The axis labels state the marker and fluorochrome used (e.g. CD4-FITC).
- [x] The axis scales are clearly visible. Include numbers along axes only for bottom left plot of group (a 'group' is an analysis of identical markers).
- [ ] All plots are contour plots with outliers or pseudocolor plots.
- [x] A numerical value for number of cells or percentage (with statistics) is provided.

Methodology

Sample preparation	Cells were washed once with PBS, detached from culture plates using TrypLE and then resuspended in appropriate volumes of PBS for flow cytometry or cell sorting. Cells were placed on ice before analysis.
Instrument	BD LSR Fortessa with HTS; Bigfoot Spectral Cell Sorter
Software	BD FACSDIVA 8.0; Bigfoot Spectral Cell Sorter Sasquatch (SQ) software
Cell population abundance	For cell sorting, around 7-10% high-GFP, around 40-55% medium-GFP, around 10-20% low-GFP population were sorted from the GFP-positive population. The sorting efficiency was estimated around 78% to 89% as determined by the cell sorter.
Gating strategy	The GFP positive gate was defined using the wild-type cells as the background (boundary of negative population). The positive gate was defined so the wild-type cells contained around 0.1% GFP-positive population.

- [x] Tick this box to confirm that a figure exemplifying the gating strategy is provided in the Supplementary Information.
