# Supplementary material for: SHIELD: a platform for high-throughput screening of barrier-type DNA elements in human cells
Source: Nat Commun. 2023 Sep 12;14:5616. doi: 10.1038/s41467-023-41468-3 (PMC10497619; doi:10.1038/s41467-023-41468-3)
Supplement: Supplementary file 4 — Description of Additional Supplementary Files [file 41467_2023_41468_MOESM4_ESM.pdf]

Title: Supplementary Data 1

Description: This file includes the information of 1000 DNA elements selected for screening, including their libarary ID (cololum 1) and correpsonding DNA sequence (column 2). Each DNA is 300 bp in length, consisting of 250 bp genomic sequence flanked by
